# Comparative Antioxidant and Antimicrobial Activities of Several Conifer Needles and Bark Extracts

**DOI:** 10.3390/pharmaceutics16010052

**Published:** 2023-12-28

**Authors:** Diana Ionela Popescu (Stegarus), Adina Frum, Carmen Maximiliana Dobrea, Ramona Cristea, Felicia Gabriela Gligor, Laura Gratiela Vicas, Roxana Elena Ionete, Nicoleta Anca Sutan, Cecilia Georgescu

**Affiliations:** 1 National Research and Development Institute for Cryogenic and Isotopic Technologies—ICSI Ramnicu Valcea, 240050 Ramnicu Valcea, Romania; diana.stegarus@icsi.ro (D.I.P.); roxana.ionete@icsi.ro (R.E.I.); 2 Preclinical Department, Faculty of Medicine, “Lucian Blaga” University of Sibiu, 550169 Sibiu, Romania; felicia.gligor@ulbsibiu.ro; 3 Department of Agricultural Sciences and Food Engineering, “Lucian Blaga” University of Sibiu, 550012 Sibiu, Romania; ramona.cristea@ulbsibiu.ro (R.C.); cecilia.georgescu@ulbsibiu.ro (C.G.); 4 Department of Pharmacy, Faculty of Medicine and Pharmacy, University of Oradea, 410028 Oradea, Romania; laura.vicas@gmail.com; 5 Department of Natural Sciences, Piteşti University Center, National University of Science and Technology Politechnica Bucharest, 110040 Pitesti, Romania; anca.sutan@upit.ro

**Keywords:** antioxidant activity, antimicrobial activity, polyphenols, Douglas fir, larch, pine, spruce, silver fir

## Abstract

Nowadays, an increased concern regarding using natural products for their health benefits can be observed. The aim of this study was to assess and compare several phenolic compounds found in 15- to 60-year-old Douglas fir, silver fir, larch, pine, and spruce needle and bark extracts and to evaluate their antioxidant and antimicrobial activities. Spectrophotometric assays were used to determine the total polyphenol content and the antioxidant activity that was assessed by using the DPPH• radical scavenging assay (RSA), the ferric reducing antioxidant power assay (FRAP), and the ABTS•+ radical cation scavenging assay (ABTS). The phytochemical content was determined by using high-performance liquid chromatography, and the antimicrobial activity was determined by assessing the minimal inhibition concentration (MIC). The results of the study show a total polyphenol content of 62.45–109.80 mg GAE/g d.w. and an antioxidant activity of 91.18–99.32% for RSA, 29.16–35.74 µmol TE/g d.w. for FRAP, and 38.23–53.57 µmol TE/g d.w. for ABTS. The greatest quantity of phenolic compound for most of the extracts was for (+)-catechin, and it had values between 165.79 and 5343.27 µg/g d.w. for these samples. The antimicrobial inhibition for all the extracts was the strongest for *Staphylococcus aureus* (MIC 62.5–125 µg/mL). The extracts analyzed could be used for their bioactive potential after further investigations.

## 1. Introduction

Worldwide, the interest in consuming natural products for their health benefits is increasing. Currently, there is great concern regarding the valorization of plants from spontaneous or cultivated flora. The study of biologically active compounds existing in different plant species arouses the interest of many researchers in terms of identification, quantification, determination of their pharmacological effects, and elucidation of their mechanisms of action [1,2].

In order for plants to be used for medicinal purposes, their phytochemical content must be known, along with the plant parts in which the bioactive compounds are present. Also of particular importance is the ability to identify, isolate, and quantify these compounds [1,2,3].

The use of phytochemicals with an antioxidant effect is constantly increasing due to their properties to provide multiple health benefits [4,5].

In the human body, protective mechanisms have been developed against damage caused by oxidation reactions. These include a variety of compounds, endogenous and exogenous, that have a synergistic action to neutralize free radicals. These components can be polyphenols from the daily diet [6,7,8].

Polyphenols are secondary metabolites that have a wide range of structures based on the phenolic ring and can be classified as flavonoids, phenolic acids, tannins, lignans, and stilbenes. In relation to their biological properties, some of the benefits that are attributed to these compounds are linked to their antioxidant, antimicrobial, anti-inflammatory, anticarcinogenic, antidiabetic, and cardioprotective and neuroprotective properties [5,9,10,11,12,13,14,15].

A review performed by Bhardwaj et al. (2021) regarding the therapeutic potential of coniferous plants revealed the traditional use of various conifers against diseases like diabetes mellitus, coronary heart disease, cancer, inflammation, and neurodegenerative diseases. Quercetin, rutin, and resveratrol extracted from conifers were reported to have sedative, antidiabetic, anticancer, and anesthetic effects. Conifer phytochemicals were reported to have beneficial properties in regulating glucose levels, insulin secretion, and lipid metabolism, as well as a protective action against reactive oxygen species (ROS), thus reducing oxidative stress. Various conifer extracts were reported to possess antimicrobial activity as well, because of their potential for degrading the cell wall of microorganisms [16].

The use of many antibiotics is affected by the antimicrobial resistance phenomenon, thus increasing morbidity and mortality all over the world [17]. The scientific world is increasingly concerned with finding viable alternatives to decrease the incidence of this phenomenon, so plant extracts are studied for their antimicrobial activities [18,19,20,21].

Coniferous plants like silver fir (*Abies alba* Mill.), spruce (*Picea abies* (L.) H. Karst.), Douglas fir (*Pseudotsuga menziesii* (Mirb.) Franco.), pine (*Pinus sylvestris* L.), and larch (*Larix decidua* Mill.) are commonly found in European forests. Their extracts can be considered a source of phytochemicals, and they can be used for the preservation of human health because of their antioxidant and antimicrobial properties [16,22,23,24,25]. Several phenolic compounds can be quantified in extracts from these conifers, such as gallic acid, caffeic acid, syringic acid, p-coumaric acid, ferulic acid, chlorogenic acid, vanillic acid, t-cinnamic acid, catechin, rutin, quercetin, apigenin, kaempherol, luteolin, etc., and they can provide antimicrobial activity against several bacteria and fungi like *Pseudomonas aeruginosa*, *Acinetobacter baumannii*, *Klebsiella pneumoniae*, *Enterococcus faecium*, *Staphylococcus aureus*, *Candida* sp., *Aspergilius* sp., etc. [21,26,27,28,29].

Nowadays, the plant extracts that are used in folk medicine have gathered the attention of pluridisciplinary research groups. Studies are performed to assess their potential for preserving and promoting human health in order to have a better understanding of their effects on the human body [16].

Based on the studies presented above, coniferous needles and bark could possess health-promoting properties due to their phytochemical composition.

As far as we know, until present, studies did not report the influence of age on phytochemical composition and antioxidant and antimicrobial activities in silver fir, spruce, Douglas fir, and pine needles and bark extracts. The importance of assessing the phytochemical composition of plants regarding their age is closely related to the proper harvesting moment of the plant material.

The aim of this study was to assess and compare several phenolic compounds found in 15- to 60-year-old coniferous needle and bark extracts and to evaluate their antioxidant and antimicrobial activities. Needles, being a fast-renewable vegetal source that can be harvested in high amounts without endangering the wellbeing of the plant, were of particular interest for this study.

## 2. Materials and Methods

### 2.1. Chemicals

The standards of caffeic acid, (+)-catechin, cinnamic acid, chlorogenic acid, ferulic acid, gallic acid, resveratrol, rutin, syringic acid, quercetin, methanol suitable for HPLC analysis, ABTS (2,2′-azinobis [3-ethylbenzothiazoline-6-sulfonic acid]-diammonium salt), DPPH (2,2-diphenyl-1-picrylhydrazyl), TPTZ (2,4,6-tripyridyl-s-triazine), Trolox (6-hydroxy-2,5,7,8-tetra-methyl-chroman-2-carboxylic acid), Folin–Ciocalteu reagent, Mueller–Hinton agar, Mueller–Hinton broth, Czapek Dox agar, and Czapek Dox broth were purchased from Sigma-Aldrich (St. Louis, MO, USA). Acetic acid, ascorbic acid, ferric chloride, hydrochloric acid, methanol, sodium acetate, and sodium carbonate (all analytical grades) were purchased from the Chemical Company (Iași, Romania). In all analyses, ultrapure water was used (conductivity 0.05 µs/cm).

### 2.2. Sample Preparation

The bark and needles of 15- and 60-year-old silver fir (*Abies alba* Mill.) and spruce (*Picea abies* (L.) H. Karst), 15- and 55-year-old Douglas fir (*Pseudotsuga menziesii* (Mirb.) Franco) and pine (*Pinus sylvestris* L.), and 15-year-old larch (*Larix decidua* Mill.) were collected in June 2021, from Romania, Valcea County, from altitudes between 1050 and 1100 m for silver fir and spruce, 500 and 550 m for Douglas fir and pine, and 850 m for larch.

The raw plant material was dried at 40 °C in airflow until it reached a constant mass and then ground on a domestic mill. Prior to the analysis, it was stored in sealed bags at room temperature, away from sunlight.

The sample voucher specimens (no. 308/1–308/18, June 2021) are held at the ”Lucian Blaga” University of Sibiu, Romania, in the Biotechnologies and Food Engineers Research Center.

### 2.3. Extraction

For this, 500 mg of each sample of powder were weighted and extracted for 30 min with 10 mL of methanol:water = 70:30 *v*/*v* solvent, introduced in an ultrasonic bath that was heated at 40 °C. After the time elapsed, the mixture was cooled and filtered, and the solvent volume was adjusted to 10 mL with the same solvent [30].

### 2.4. Analysis

#### 2.4.1. Determination of the Total Polyphenolic Content (TPC)

In a test tube, 0.4 mL of sample solution, 1 mL of Folin–Ciocalteu reagent, 15 mL of water, and 2 mL of a 290 g/L Na_2_CO_3_ solution were added. The mixture was shaken for 10 min and kept for 20 min in a water bath at 40 °C. After the time elapsed, the mixture was cooled, and the extinction was recorded by using an UV 1900 spectrophotometer (Shimadzu, Kyoto, Japan) at λ = 760 nm [31].

A linear calibration curve (9–45 µg gallic acid/mL) was performed, and the results were expressed as mg GAE/g d.w.

#### 2.4.2. Determination of the DPPH• Radical Scavenging Assay (RSA)

In a volumetric flask, a DPPH stock solution was prepared (25 µg DPPH/mL methanol). After 2 h in which the stock solution was kept in the dark at a low temperature, 970 µL of it was mixed with 30 µL of sample solution. The extinction was recorded by using a Shimadzu UV 1900 spectrophotometer at λ = 515 nm, and the results were expressed as (%) [30,32]. A 1 mg/mL ascorbic acid solution was used as a control. A linear calibration curve was performed (0.25–250 µg DPPH/mL), and the results were calculated by using the following formula:

$$\mathrm{RSA}\;(\%)=\frac{C_{ss}-C_{sa}}{C_{ss}}\cdot 100,$$

where RSA = radical scavenging activity for DPPH (%), C_ss_ = stock solution concentration (µg DPPH/mL), and C_sa_ = sample concentration (µg DPPH/mL).

#### 2.4.3. Determination of the Ferric-Reducing Antioxidant Power (FRAP)

The FRAP assay used was applied according to the method proposed by Vicas et al. (2015), with some modifications. Briefly, to obtain the FRAP solution, 50 mL of 300 mM (pH = 3.6) acetate buffer were mixed with 5 mL of FeCl_3_ (20 mM), and 5 mL of TPTZ solution (10 mM) acidified with 150 µL HCl was added to it. In a test tube, 0.1 mL of extract, 0.5 mL of FRAP solution, and 2 mL of purified water were introduced and kept in the dark for 1 h prior to the analysis. After the time elapsed, the extinction was recorded by using a Shimadzu UV 1900 spectrophotometer at λ = 595 nm [33].

A linear calibration curve (0.15–0.5 µmol Trolox/mL) was performed, and the results were expressed as µmol TE/g d.w.; the positive control was ascorbic acid.

#### 2.4.4. Determination of the ABTS•+ Radical Cation Scavenging Assay (ABTS)

The ABTS assay used was applied according to the method proposed by Vicas et al. (2015), with some modifications. Briefly, a stock solution of potassium persulfate (2.45 mM) and 2,2′-azinobis [3-ethylbenzothiazoline-6-sulfonic acid]-diammonium salt (7 mM) were mixed and kept in the dark at room temperature for 16 h. After the time elapsed, the stock solution was diluted, so the extinction (λ = 734 nm) would be 0.70 ± 0.02. In a test tube, 20 µL of extract was added along with 2 mL of diluted stock solution and mixed using a vortex for 30 s. After exactly one minute, the extinction was registered by using a Shimadzu UV 1900 spectrophotometer at λ = 734 nm [33].

A linear calibration curve (0.125–2.0 µmol Trolox/mL) was performed, and results were expressed as µmol TE/g d.w.; the positive control was ascorbic acid.

#### 2.4.5. Determination of Phenolic Compounds by HPLC-UV Assay

The HPLC-UV method for the identification and quantification of phenolic compounds was determined by using methods that were already conducted on plants and dietary supplements [31,34]. The analysis was carried out using a SCL-40 HPLC system (Shimadzu, Kyoto, Japan) equipped with a degasser, quaternary pump, photodiode array detector, thermostatic column oven, and autosampler. The used column was Nucleosil C18 (250 mm × 4.6 mm, i.d. 5 µm) at a temperature of 25 °C. The used mobile phases were: A: purified water, B: methanol, and C: purified water: acetic acid (96:4 (*v*/*v*)). The gradient was: at 0 min: 15% B and 85% C, at 15 min: 75% A and 25% B, at 20 min: 15% A and 85% B, at 40 min: 40% A and 60% B, and column conditioning. The injection volume was 5 µL. For the first 15 min, the flow rate was 0.5 mL/min and 0.8 mL/min for the rest of the analysis. Rutin and quercetin were detected at λ = 360 nm, caffeic acid, chlorogenic acid, and ferulic acid at λ = 330 nm, resveratrol at λ = 306 nm, and (+)-catechin, cinnamic acid, and syringic acid at λ = 280 nm. The identification and quantification of the phenolic compounds analyzed were performed using standard phenolic compounds (Figures S1 and S2). The purities of the used standard phenolic compounds were: ≥98% for (+)-catechin, ≥95% for syringic acid, ≥99% for cinnamic acid, ≥99% for resveratrol, ≥99% for caffeic acid, ≥99% for chlorogenic acid, ≥99% for ferulic acid, ≥94% for rutin, and ≥95% for quercetin.

#### 2.4.6. Determination of Antimicrobial Activity

The obtained extracts were centrifuged and concentrated through solvent evaporation so that the methanol from the solvent would not influence the assay.

The following pathogens were used for this assay: Gram-positive bacteria: *Staphylococcus aureus* ATCC29213, *Bacillus subtilis* ATCC23857, *Streptococcus pyogenes* ATCC19615, Gram-negative bacteria: *Escherichia coli* ATCC25922, *Pseudomonas aeruginosa* ATCC10145, *Morganella morganii* ATCC25830, and pathogen yeasts: *Candida albicans* ATCC14053, *Candida parapsilosis* ATCC22019, as they are frequently incriminated in human infectious diseases [35,36,37,38].

The modified Kirby-Bauer diffusion method was performed to assess the antimicrobial activity of the analyzed samples. Briefly, 10 mL of Mueller–Hinton broth/Czapek Dox broth culture medium was used for the 24 h activation of 100 µL of the test bacteria and yeast. Mueller–Hinton agar/Czapek Dox agar culture medium was used for the preparation of the Petri dishes, which were inoculated with 100 µL of 0.5 Mc Farland (density 0.5 McF = 1.5 × 108 CFU/mL) bacterial suspension. Dry extracts were weighed and dissolved in ultrapure water to final concentrations of 1000 µg/mL (stock) and then tested by using sterilized filter paper discs (6 mm diameter). Then, 10 µL test samples were used for disc impregnation, which were incubated at 37 °C for 24/48 h. The inhibition zone diameters (mm) were measured with a ruler. The mean of the three determinations performed was reported [39].

*Determination of the Minimal Inhibition Concentration*
*Antibacterial activity*

For the assessment of the minimal inhibition concentration against 50% of the microbial strains analyzed (MIC), the disc diffusion method with successive dilutions was used. Briefly, the plant extracts (stock) were diluted to the following values: from 1000 µg/mL to 750 µg/mL, 500 µg/mL, 250 µg/mL, 125 µg/mL, and 62.5 µg/mL. Discs impregnated with 10 µL plant extract dilutions were placed in a solidified Mueller–Hinton culture medium that contained the microorganism of interest and were incubated for 24 h at a temperature of 37 °C. After the time elapsed, the zone of inhibition was measured, and the results were interpreted. The MIC was considered the extract’s lowest concentration that could inhibit bacterial growth. The positive control used was gentamicin [39].

*Antifungal activity*

Plant extracts were concentrated to a value of 1000 µg/mL in dimethyl sulfoxide (DMSO), and then five successive dilutions were performed, resulting in final concentrations with the following values: 750 µg/mL, 500 µg/mL, 250 µg/mL, 125 µg/mL, and 62.5 µg/mL. The activation of the yeast strains was performed in a Czapek Dox liquid culture medium for 24 h at 25 °C. The concentration was adjusted spectrophotometrically to 0.5 McFarland. Then, 6 mm discs impregnated with 10 µL plant extract dilutions were placed in a Czapek Dox agar culture medium containing yeast strains and incubated for 72 h at a temperature of 25 °C. After the time elapsed, the zone of inhibition was measured, and the results were interpreted. The MIC was considered the extract’s lowest concentration that could inhibit yeast growth. The positive control used was ketoconazole [39].

#### 2.4.7. Statistical Analysis

In order to assess the statistical significance of the obtained results, the analyses were performed in triplicate (*n* = 3), and the results were expressed as mean ± standard deviation (SD). The software used for the statistical analysis of the obtained results was IBM SPSS Statistics version 20 (SPSS Inc., Chicago, IL, USA), and the tests performed were one-way ANOVA (analysis of variance) with post hoc Tukey’s test (significance level: *p* < 0.05).

## 3. Results

### 3.1. Phytochemical Content and Antioxidant Activity

As indicated in Table 1, all the conifer samples presented quantifiable total polyphenol content (TPC) and thus antioxidant activities as well. The highest TPC was determined for needles of 15-year-old spruce (SP1N) in a quantum of 109.80 ± 1.39 mg gallic acid equivalents (GAE)/g sample dry weight (d.w.), followed by needles of 15-year-old Douglas fir (DO1N) and bark of 15-year-old spruce (SP1B) that had quantities greater than 103.6 mg GAE/g d.w.

The registered data shows a decrease in the TPC from 15 to 55–60 years of age for spruce needles and bark (SPN and SPB), Douglas fir needles (DON), and pine needles and bark (PIN and PIB) and an increase in the TPC for silver fir needles and bark (FIN and FIB) and Douglas fir bark (DOB). The needles from the analyzed coniferous samples presented higher quantities of total polyphenols when compared to the bark of each sample for spruce (SP), Douglas fir (DO), pine (PI), and larch (LA), and slightly lower quantities for silver fir (FI).

The antioxidant activity of the analyzed coniferous samples obtained by using the RSA was between 99.32 and 91.18%, while the antioxidant activity of a 1 mg/mL ascorbic acid solution was 100.00%. The highest values obtained were for FI2N (99.32 ± 1.94%), followed by PI2N (99.22 ± 1.58%), as shown in Table 1.

The ABTS•+ radical cation scavenging assay (ABTS) was used for the assessment of the antioxidant activity of the samples as well. The results show that the values obtained were between 38.23 ± 1.13 and 53.57 ± 1.27 µmol Trolox equivalents (TE)/g d.w.

Regarding the FRAP assay, the results obtained show antioxidant activities between 35.74 and 29.16 µmol TE/g d.w. The highest value obtained was for FI2N (35.74 ± 1.20 µmol TE/g d.w.).

The antioxidant activities assessed for ascorbic acid expressed as mmol TE/g had much higher values than the results obtained for the analyzed plant extracts.

An increase in the antioxidant activity of the samples analyzed from 15 to 55–60 years was registered for FIN, FIB, and DOB, as shown for the TPC assay as well. All the samples showed a decrease in antioxidant activity in barks compared to needles for each coniferous analyzed.

The results presented in Table 1 show that the antioxidant activity is higher in the needle samples than in the bark samples. These results can be correlated with the TPC, except for the FI samples, where FIB had a higher TPC than FIN and FIN had higher antioxidant activity than FIB. This may occur due to other compounds that have antioxidant potential but are not polyphenols.

As shown in Table 2, quercetin was determined for all the analyzed samples and had values between 687.69 ± 1.82 µg/g d.w. for LA1B and 32.90 ± 0.83 µg/g d.w. for FI1B. Syringic acid, (+)-catechin, rutin, and resveratrol were quantified in nearly all the samples analyzed, except PIB samples for syringic acid, DO1B samples for (+)-catechin, and SPB samples for rutin and resveratrol. Caffeic acid was quantified only for FIN samples, and cinnamic acid and ferulic acid were found only in the needle samples, except for LA and DO, respectively, in which they were identified in the bark as well.

For 13 of the 18 samples analyzed, the greatest quantity of phenolic compound was (+)-catechin, and it had values between 5343.27 and 165.79 µg/g d.w. for these samples. The greatest quantity of phenolic compound for SP1N was 1498.90 ± 1.20 µg syringic acid/g d.w., for DO1B was 2946.50 ± 1.06 µg ferulic acid/g d.w., for LA1N was 1115.85 ± 1.56 µg ferulic acid/g d.w., for DO1N was 576.90 ± 0.99 µg rutin/g d.w., and for LA1B 687.69 ± 1.82 µg quercetin/g d.w.

The results show that in all the samples analyzed, the needles showed a greater variety of identified compounds that the bark and the age of the coniferous did not affect the composition of the phytochemicals analyzed, except for DO1B, in which (+)-catechin was not identified, even though it was present in the DO2B sample.

A quantity decrease in phenolic compounds and derivatives with age could be identified for cinnamic acid in SPN, FIN, and PIN; syringic acid in SPB and DOB; chlorogenic acid in SPB; ferulic acid in DOB and PIN; (+)-catechin in SPN, FIN, FIB, PIN, and PIB; rutin in SPN, FIB, PIN, and PIB; quercetin in SPN, FIB, DOB, PIN, and PIB; and resveratrol in SPN, FIN, and PIN.

### 3.2. Antimicrobial Activity

As presented in Table 3, SP extracts showed strong and very strong antibacterial activity against the bacterial strain *Staphylococcus aureus* ATCC 29213, with the minimal inhibitory concentration (MIC) being between 62.5 and 125 µg/mL. The Gram-positive bacteria *Bacillus subtilis* ATCC 23857 reacted only to a small or very small extent to SP extracts, with the MIC values being between 500 and 750 µg/mL. This group of Gram-positive bacteria also includes *Streptococcus pyogenes* ATCC 19615, which was inhibited by SP extracts and showed low to medium antibacterial activity, with the resulting MIC values being between 250 and 500 µg/mL.

For the Gram-negative bacteria, the most significant results were obtained in the case of the *Escherichia coli* strain ATCC 25922, where the extracts from SP showed strong antibacterial activity (with an MIC value of 125 µg/mL). The antibacterial activity was also shown against *Pseudomonas aeruginosa* ATCC10145 to be medium to low, with the MIC value being between 250 and 500 µg/mL. In the case of the *Morganella morganii* strain ATCC 25830, the activity was very low to inexistent.

The results observed in the case of the yeast strain *Candida albicans* ATCC 14053, where the MIC was between 125 and 250 µg/mL, were that the antifungal activity was medium to strong; on the other hand, the SP extracts presented an activity that was very low to none for the *Candida parapsilosis* ATCC22019 strain.

Regarding the FI extracts, a very strong to strong activity was observed against the Gram-positive strain *Staphylococcus aureus* ATCC 29213, with MIC being between 62.5 and 125 µg/mL; absent against *Bacillus subtilis* ATCC23857; and medium to strong activity against *Streptococcus pyogenes* ATCC19615, with MIC values between 125 and 250 µg/mL. For the Gram-negative bacteria *Escherichia coli*, the antibacterial activity of FI extracts was very strong to moderate (MIC values between 62.5 and 250 µg/mL); FI2N and FI2B had MIC values of 62.5 µg/mL, thus possessing very strong antibacterial activity. A moderate activity was observed against the Gram-negative strain *Pseudomonas aeruginosa* ATCC10145 (with an MIC value of 250 µg/mL) and a very low activity (with an MIC value of 750 µg/mL) or non-existent one against M. morganii ATCC25830. A strong activity was observed against the *Candida albicans* ATCC 14053 yeast strain, where the MIC was 125 µg/mL. A very low to no activity was observed against the strain *Candida parapsilosis* ATCC22019, with the MIC being 750 µg/mL or more.

The extracts from DO, PI, and LA showed very strong to strong antibacterial activity against *Staphylococcus aureus* ATCC29213, with the MIC being between 62.5 and 125 µg/mL; low to very low activity for *Bacillus subtilis* ATCC23857 (MIC value between 500 and 750 µg/mL); strong to low activity against Gram-positive *Streptococcus pyogenes* ATCC19615 (MIC value between 125 and 500 µg/mL); and strong to medium activity against Gram-negative *Escherichia coli* ATCC25922 (MIC value between 125 and 250 µg/mL). A strong to low antibacterial activity was observed against *Pseudomonas aeruginosa* ATCC10145 (MIC value between 125 and 500 µg/mL). The antifungal activity of DO extracts was strong to medium against *Candida albicans* ATCC14053 (MIC value between 125 and 250 µg/mL), and PI and LA extracts had a strong activity against this yeast, with a MIC value of 125 µg/mL. The antifungal activity against *Candida parapsilosis* ATCC22019 was inexistent for DO extracts, and for PI and LA extracts, it was very low to no action, with MIC values of 750 µg/mL and more.

## 4. Discussion

The analyzed vegetal products are rich in polyphenols (Table 1), but quantitative variations could be observed between the samples. By comparing the amount of quantified phytochemical compound (Table 2) with the conifer part (needles or bark) considering the age as well, we could observe that regarding the SP samples, they had the greater amounts of syringic acid and chlorogenic acid in the bark samples and (+)-catechin and quercetin in SP2B and SP1N. The FI samples presented the highest amounts of syringic acid, (+)-catechin, and quercetin in the needle samples, resveratrol in the bark sample, and rutin in FI2N and FI1B. Regarding the DO samples, the greatest amounts of resveratrol and rutin were found in the needle samples, as were syringic acid, ferulic acid, and quercetin in DO2N and DO1B. The greatest amounts of phytochemicals in the PI samples were found in the needles for resveratrol and rutin, PI1N and PI2B for (+)-catechin, and PI2N and PI1B for quercetin. The LA samples showed larger quantities of cinnamic acid and quercetin in the bark samples and syringic acid, (+)-catechin, rutin, and resveratrol in the needles.

Different results were reported by other studies performed on conifers in different parts of the world. These variations may be the result of cumulative factors such as soil composition, climacteric conditions, extraction method, etc. The study by Dziedzinski et al. (2020) showed high antioxidant activity for SP, PI, DO, and LA. Comparing with the results obtained in this study, higher quantities of syringic acid for PI and LA (145.44 µg/g d.w. and 13.15 µg/g d.w., respectively) and lower quantities for DO and SP (113.97 µg/g d.w. and 301.96 µg/g d.w., respectively) were obtained in the mentioned study. Rutin and quercetin had lower quantities in SP, PI, DO, and LA (0.31–1.14 µg/g d.w. for rutin and 0.63–1.38 µg/g d.w. for quercetin) [26].

Bhardwaj et al. (2021) reported several constituents present in conifers, amongst other phytochemicals: quercetin, rutin, (+)-catechin, caffeic acid, cinnamic acid, syringic acid, chlorogenic acid, and t-resveratrol, which were identified in SP, PI, DO, and LA [16]. Also, the antioxidant activity and the total polyphenolic content were confirmed for all the extracts analyzed in this study [16,25].

Szwajkowska-Michałek et al. (2020) quantified several phenolic acids for PI, FI, LA, and SP needle extracts and showed lower quantities of caffeic acid for the FI extracts (between 27.4 and 30.8 µg/g d.w.). The syringic acid content was lower than the one obtained in this study, and it had values between 3.6 and 4.3 µg/g d.w. for FI, 15.9–16.3 µg/g d.w. for LA, 5.3–8.9 µg/g d.w. for PI, and 18.3–20.1 µg/g d.w. for SP. Regarding ferulic acid, lower values were obtained for FI (0.5–1.1 µg/g d.w.), LA (6.4–7.4 µg/g d.w.), and SP (52.4–70.2 µg/g d.w.), and higher values were observed for PI (88.6–109.9 µg/g d.w.). Comparable values were obtained for cinnamic acid and chlorogenic acid regarding all the extracts analyzed, with the exception of chlorogenic acid for LA, which was not detected in this study, and for PI, for which lower values were determined in this study [27].

ROS are linked to several diseases, like diabetes mellitus, cardiovascular diseases, cancer, inflammation, and neurological conditions. Polyphenols are natural compounds that have antioxidant activity against ROS; thus, they can be linked to the prevention of ROS-related diseases [16].

All the analyzed phytochemicals present bioactive potential, as reported by several studies. A review provided by Stromsnes et al. (2021) presented studies regarding several of the analyzed phenolic compounds. Thus, resveratrol intake improved the total antioxidant status, decreased the deterioration from Alzheimer’s disease, decreased the C-reactive protein concentrations, and, along with quercetin, decreased blood pressure. The consumption of resveratrol and flavones reduced the risk of Chron’s disease. Quercetin showed apoptotic effects, and quercetin, rutin, and chlorogenic acid decreased the symptoms of colitis. Catechin was studied for its apoptotic effects in breast, pancreatic, and colorectal cancers, and chlorogenic acid reduced neurodegeneration and provided neuroprotection against Parkinson’s disease [40,41]. The antioxidant potential of stilbenes (i.e., resveratrol) was assessed by Reinisalo et al. (2015), who revealed their therapeutic potential of reducing oxidative stress, thus decreasing the incidence of age-related diseases [42]. Rutin was reported to have beneficial effects in the management of neurodegenerative and cardiovascular disorders due to its antioxidant and anti-inflammatory activities [43,44]. Due to its antioxidant potential, ferulic acid could exert several effects, such as antidiabetic, anticancer, anti-aging, neuroprotective, and cardioprotective [45]. Chlorogenic, caffeic, syringic, cinnamic, and ferulic acids are associated with antioxidant and antimicrobial effects and can reduce the risk of several diseases, like cardiovascular and neurodegenerative diseases and type 2 diabetes mellitus [46,47,48].

Dziedzinski et al. (2020) presented the antibacterial activity of extracts of SP, PI, DO, and LA. The inhibition of *P. aeruginosa* for the LA extract was very low; for the extract of DO, it was low; for the PI extract, it was strong; and for the extract of SP, it was very strong in comparison with the results of this study. In this study, for the LA extracts, the inhibition was medium to strong; for DO extracts, it was low to medium; for PI extracts, it was medium; and for SP, it was medium to low. The *S. aureus* inhibition results obtained in the mentioned study could be compared to the results of this study; so, for the LA extract, *S. aureus* inhibition was low; for the extract of DO, it was not existent; for the PI extract, it was medium; and for the extract of SP, it was strong. In this study, for all the extracts analyzed, the inhibition was strong to very strong [26].

There are many microorganisms involved in human pathology that can be inhibited with the help of extracts or essential oils obtained from various conifers. Thus, the study carried out by Garzoli et al. (2021) on the needles of *Pinus cembra* L., *Pinus mugo* Turra, *Picea abies* L., and *Abies alba* Mill. highlighted the antibacterial action of Gram-negative bacteria, like *Escherichia coli*, *Pseudomonas fluorescens,* and *Acinetobacter bohemicus,* and Gram-positive bacteria, like *Kocuria marina* and *Bacillus cereus*, with the MIC results being between 12.82 mg/mL and 53.12 mg/mL [49].

The antibacterial activity of hydro-methanolic extracts from *Pinus cembra* L. was studied by Apetrei et al. (2011) on Gram-positive bacteria like *Staphylococcus aureus*, *Sarcina lutea*, and *Bacillus cereus*; Gram-negative bacteria like *Escherichia coli* and *Pseudomonas aeruginosa*; and pathogenic yeasts like *Candida albicans*, with results demonstrating that both bark and needles are rich in valuable compounds with antioxidant and antimicrobial potential [50].

Hydroalcoholic extracts from the bark of *Larix decidua* Mill. showed strong antibacterial activity, which was evaluated in the work published by Faggian et al. (2021) on pathogenic bacterial strains commonly encountered in the respiratory tract, namely *Staphylococcus aureus*, *Streptococcus pyogenes*, *Streptococcus pneumoniae*, *Klebsiella pneumoniae*, *Pseudomonas aeruginosa*, and *Haemophilus influenza* [51].

## 5. Conclusions

The extracts of silver fir (*Abies alba* Mill.), spruce (*Picea abies* (L.) H. Karst), Douglas fir (*Pseudotsuga menziesii* (Mirb.) Franco), pine (Pinus sylvestris L.), and larch (*Larix decidua* Mill.) presented comparable antioxidant potential to ascorbic acid for the DPPH• radical scavenging assay and lower ferric-reducing antioxidant power and ABTS•+ radical cation scavenging power than ascorbic acid. The greatest quantity of phenolic compounds in most of the extracts was for (+)-catechin, and most of the compounds quantified were for the needle samples compared to the bark samples.

A quantity decrease in phenolic compounds and derivatives from 15 to 55–60 years of age could be identified in the analyzed samples for the cinnamic acid in SPN, FIN, and PIN; syringic acid in SPB and DOB; chlorogenic acid in SPB; ferulic acid in DOB and PIN; (+)-catechin in SPN, FIN, FIB, PIN, and PIB; rutin in SPN, FIB, PIN, and PIB; quercetin in SPN, FIB, DOB, PIN, and PIB; and resveratrol in SPN, FIN, and PIN.

The antimicrobial inhibition for all the extracts was the strongest for *Staphylococcus aureus.*

Studies regarding the mechanism of action of bioactive compounds and the efficacy and toxicity of the needles and bark extracts of the analyzed conifers should be performed in order to assess their health-promoting properties.

In comparison to the bark, needles have the added benefit of being a highly renewable and accessible vegetal product.

## Figures and Tables

**Table 1:** Antioxidant activity and total polyphenolic content of the analyzed extracts.

Sample	TPC mg GAE/g d.w.	RSA %	FRAP µmol TE/g d.w.	ABTS µmol TE/g d.w.
SP1N	109.80 ± 1.39 ^a^	98.07 ± 1.38 ^a^	34.93 ± 1.42 ^a,b^	50.83 ± 0.55 ^a,f^
SP2N	102.59 ± 1.54 ^a,d^	96.50 ± 1.90 ^a,h^	31.94 ± 1.35 ^a,l^	48.99 ± 1.05 ^c–h,k^
SP1B	103.61 ± 1.01 ^a,c,e^	97.65 ± 1.23 ^a,c^	34.49 ± 1.31 ^a,f^	50.71 ± 0.90 ^a,g^
SP2B	101.38 ± 10.02 ^b–e^	92.43 ± 1.93 ^c–i^	31.71 ± 2.02 ^a,m^	42.58 ± 0.95 ^m^
FI1N	81.56 ± 1.05 ^i–k^	98.90 ± 1.69 ^a^	34.13 ± 1.33 ^a,g^	52.91 ± 1.56 ^a,b^
FI2N	85.92 ± 1.41 ^j,k^	99.32 ± 1.94 ^a^	35.74 ± 1.20 ^a^	53.57 ± 1.27 ^a^
FI1B	88.11 ± 1.32 ^i,h^	95.46 ± 1.86 ^a,i^	33.14 ± 1.05 ^a,i^	46.52 ± 0.66 ^i–l^
FI2B	93.28 ± 1.12 ^f–h^	98.59 ± 2.04 ^a^	34.69 ± 1.28 ^a,d^	51.73 ± 1.06 ^a,d^
DO1N	103.64 ± 1.17 ^a,b^	97.75 ± 1.55 ^a,b^	32.87 ± 0.94 ^a,k^	50.33 ± 0.91 ^a,h^
DO2N	100.76 ± 1.22 ^b–d,f^	97.44 ± 1.88 ^a,d^	33.06 ± 1.62 ^a,j^	50.19 ± 1.01 ^b–h^
DO1B	75.25 ± 1.13 ^k,l^	91.80 ± 1.93 ^h,i^	29.46 ± 1.01 ^h–m^	39.80 ± 0.73 ^m,o^
DO2B	84.26 ± 1.20 ^i,j^	92.74 ± 1.62 ^b–i^	29.64 ± 1.03 ^h–m^	41.36 ± 1.02 ^m,n^
PI1N	99.40 ± 0.80 ^b–d,g^	98.90 ± 1.51 ^a^	34.66 ± 0.90 ^a,e^	51.99 ± 1.87 ^a,c^
PI2N	87.60 ± 1.05 ^h,j^	99.22 ± 1.58 ^a^	33.49 ± 1.27 ^a,h^	51.65 ± 0.89 ^a,e^
PI1B	68.49 ± 1.47 ^l,m^	97.44 ± 1.88 ^a,e^	30.61 ± 1.83 ^d–g,i–m^	50.16 ± 0.88 ^b–h^
PI2B	67.12 ± 1.19 ^m^	97.44 ± 1.87 ^a,f^	29.16 ± 1.64 ^i–m^	49.17 ± 1.33 ^c–i,l^
LA1N	66.80 ± 0.79 ^m^	97.23 ± 1.13 ^a,g^	34.77 ± 1.06 ^a,c^	49.08 ± 0.90 ^c–h,j,l^
LA1B	62.45 ± 1.19 ^m^	91.18 ± 1.85 ^i^	31.29 ± 1.15 ^b–m^	38.23 ± 1.13 ^o,n^
Ascorbic acid	-	100.00 ± 0.53% *	20.17 ± 1.10 mmol TE/g d.w.	22.59 ± 1.39 mmol TE/g d.w.

TPC: total polyphenol content; RSA: DPPH• radical scavenging assay; FRAP: ferric-reducing antioxidant power; ABTS: ABTS•+ radical cation scavenging assay; GAE: gallic acid equivalents; TE: Trolox equivalents; SP: spruce; FI: silver fir; DO: Douglas fir; PI: pine; LA: larch; 1: 15 years old; 2: 55–60 years old; N: needles; B: bark; *: ascorbic acid solution 1 mg/mL. Results were performed in triplicate (*n* = 3) and expressed as the mean ± standard deviation (SD). Results from the same column followed by the same letters do not present a significant difference (*p* < 0.05).

**Table 2:** Phytochemical composition.

	Phytochemical Compound µg/g d.w.								
Sample	Phenolic Acids and Derivatives					Flavonoids			Stilbenes
	Cinnamic Acid	Syringic Acid	Caffeic Acid	Chlorogenic Acid	Ferulic Acid	(+)-Catechin	Rutin	Quercetin	Resveratrol
SP1N	40.29 ± 0.87 ^d^	169.47 ± 1.06 ^f^	n.d.	102.78 ± 1.03 ^d^	153.90 ± 0.83 ^e^	5343.27 ± 0.89 ^a^	133.51 ± 1.18 ^e^	279.45 ± 1.22 ^e^	33.40 ± 0.76 ^e^
SP2N	12.49 ± 0.93 ^g^	554.40 ± 0.80 ^c^	n.d.	467.89 ± 1.55 ^c^	272.12 ± 1.20 ^d^	1248.80 ± 1.29 ^g^	86.86 ± 1.17 ^h^	90.23 ± 1.27 ^m^	8.75 ± 0.79 ^i^
SP1B	n.d.	1498.90 ± 1.20 ^a^	n.d.	1331.76 ± 1.54 ^a^	n.d.	981.35 ± 1.77 ^i^	n.d.	135.69 ± 1.28 ^i^	n.d.
SP2B	n.d.	809.56 ± 0.90^b^	n.d.	729.88 ± 1.99 ^b^	n.d.	2174.86 ± 1.21 ^c^	n.d.	158.09 ± 1.29 ^g^	n.d.
FI1N	54.93 ± 1.02 ^c^	154.71 ± 1.38 ^g^	79.58 ± 1.47 ^a^	8.38 ± 0.59 ^f^	88.38 ± 1.39 ^g^	3735.62 ± 1.41 ^b^	79.70 ± 1.82 ^i^	132.75 ± 1.41 ^i^	26.51 ± 0.91 ^f^
FI2N	22.22 ± 0.66 ^f^	257.28 ± 1.33 ^e^	141.76 ± 1.64 ^b^	37.53 ± 1.28 ^e^	123.42 ± 1.12 ^f^	1910.48 ± 1.38 ^d^	134.02 ± 1.46 ^e^	167.29 ± 1.78 ^f^	21.80 ± 1.07 ^g^
FI1B	n.d.	66.79 ± 1.51 ^j^	n.d.	n.d.	n.d.	852.91 ± 1.22 ^j^	114.92 ± 1.10 ^f^	127.34 ± 1.75 ^j^	26.62 ± 0.72 ^f^
FI2B	n.d.	74.12 ± 1.02 ^i^	n.d.	n.d.	n.d.	259.61 ± 0.87 ^m^	112.08 ± 1.29 ^f^	32.90 ± 0.83 ^o^	27.05 ± 1.08 ^f^
DO1N	25.96 ± 1.14 ^e^	27.60 ± 1.07 ^n^	n.d.	n.d.	59.05 ± 0.75 ^h^	491.01 ± 1.65 ^l^	576.90 ± 0.99 ^c^	39.62 ± 1.46 ^n^	89.78 ± 1.29 ^c^
DO2N	94.59 ± 0.99 ^b^	439.46 ± 1.07 ^d^	n.d.	n.d.	581.13 ± 1.59 ^c^	1094.17 ± 1.60 ^h^	927.90 ± 0.92 ^a^	428.11 ± 0.51 ^c^	279.92 ± 1.35 ^a^
DO1B	n.d.	102.89 ± 1.46 ^h^	n.d.	n.d.	2946.50 ± 1.06 ^a^	n.d.	57.72 ± 1.03 ^m^	315.35 ± 0.87 ^d^	16.94 ± 1.49 ^h^
DO2B	n.d.	35.68 ± 0.85 ^m^	n.d.	n.d.	40.60 ± 0.84 ^i^	165.79 ± 1.40 ^o^	105.43 ± 1.05 ^g^	122.26 ± 1.10 ^k^	27.43 ± 1.01 ^f^
PI1N	9.30 ± 0.94 ^h^	50.53 ± 0.65 ^l^	n.d.	4.10 ± 0.42 ^g^	42.23 ± 1.59 ^i^	1461.85 ± 1.26 ^e^	823.18 ± 1.48 ^b^	127.27 ± 1.18 ^j^	133.66 ± 1.91 ^b^
PI2N	8.56 ± 0.49 ^h^	61.49 ± 0.77 ^k^	n.d.	4.07 ± 0.36 ^g^	1.49 ± 0.39 ^j^	110.63 ± 0.60 ^p^	63.34 ± 1.32 ^k,l^	109.02 ± 1.42 ^l^	21.95 ± 0.83 ^g^
PI1B	n.d.	n.d.	n.d.	n.d.	n.d.	1378.05 ± 1.50 ^f^	76.58 ± 0.86 ^i^	151.72 ± 1.24 ^n^	10.17 ± 1.25 ^i^
PI2B	n.d.	n.d.	n.d.	n.d.	n.d.	240.15 ± 0.91 ^n^	60.43 ± 0.67 ^l,m^	42.34 ± 1.51 ^n^	17.69 ± 1.00 ^h^
LA1N	4.34 ± 1.15 ^i^	104.42 ± 1.06 ^h^	n.d.	n.d.	1115.85 ± 1.50 ^b^	516.01 ± 1.56 ^k^	354.38 ± 1.74 ^d^	532.31 ± 1.28 ^b^	56.96 ± 1.26 ^d^
LA1B	139.62 ± 1.90 ^a^	35.78 ± 1.05 ^m^	n.d.	n.d.	n.d.	88.83 ± 0.75 ^q^	71.63 ± 1.94 ^j^	687.69 ± 1.82 ^a^	18.22 ± 1.11 ^h^

SP: spruce; FI: silver fir; DO: Douglas fir; PI: pine; LA: larch; 1: 15 years old; 2: 55–60 years old; N: needles; B: bark. Results were performed in triplicate (*n* = 3) and expressed as the mean ± standard deviation (SD). Results from the same column followed by the same letters do not present a significant difference (*p* < 0.05). n.d. = not detected.

**Table 3:** Antibacterial and antifungal activity of the coniferous samples—MIC (µg/mL).

Sample	*Staphylococcus aureus* ATCC29213		*Bacillus subtilis* ATCC23857		*Streptococcus pyogenes* ATCC19615		*Escherichia coli* ATCC25922		*Pseudomonas aeruginosa* ATCC10145		*Morganella morganii* ATCC25830		*Candida albicans* ATCC14053		*Candida parapsilosis* ATCC22019	
	AA	MIC	AA	MIC	AA	MIC	AA	MIC	AA	MIC	AA	MIC	AA	MIC	AA	MIC
SP1N	++++	125	+	750	++	500	++++	125	+++	250	+	750	+++	250	+	750
SP2N	++++	125	+	750	+++	250	++++	125	++	500	−	−	+++	250	−	−
SP1B	+++++	62.5	++	500	++	500	++++	125	+++	250	+	750	++++	125	+	750
SP2B	+++++	62.5	++	500	+++	250	++++	125	+++	250	−	−	++++	125	−	−
FI1N	++++	125	−	−	+++	250	+++	250	+++	250	+	750	++++	125	+	750
FI2N	++++	125	−	−	+++	250	+++++	62.5	+++	250	−	−	++++	125	+	750
FI1B	+++++	62.5	−	−	+++	250	++++	125	+++	250	+	750	++++	125	−	−
FI2B	+++++	62.5	−	−	++++	125	+++++	62.5	+++	250	−	−	++++	125	+	750
DO1N	+++++	62.5	++	500	+++	250	+++	250	++	500	+	750	++++	125	−	−
DO2N	+++++	62.5	+	750	+++	250	+++	250	+++	250	−	−	+++	250	−	−
DO1B	++++	125	++	500	++++	125	++++	125	+++	250	+	750	+++	250	−	−
DO2B	++++	125	+	750	+++	250	++++	125	+++	250	−	−	+++	250	−	−
PI1N	++++	125	+	750	++	500	+++	250	+++	250	+	750	++++	125	+	750
PI2N	++++	125	+	750	+++	250	+++	250	+++	250	−	−	++++	125	+	750
PI1B	+++++	62.5	+	750	++++	125	++++	125	+++	250	+	750	++++	125	+	750
PI2B	++++	62.5	+	750	++++	125	++++	125	+++	250	−	−	++++	125	+	750
LA1N	++++	125	++	500	+++	250	+++	250	++	500	−	−	++++	125	−	−
LA1B	+++++	62.5	++	500	+++	250	++++	125	++++	125	−	−	++++	125	+	750
Gentamicin	+++++	62.5	++	500	++++	125	++++	125	++++	125	+++	250	−	−	−	−
Ketoconazole	−	−	−	−	−	−	−	−	−	−	−	−	+++++	62.5	+++++	62.5

SP: spruce; FI: silver fir; DO: Douglas fir; PI: pine; LA: larch; 1: 15 years old; 2: 55–60 years old; N: needles; B: bark. AA: antibacterial and antifungal activity; MIC: minimal inhibitory concentration (µg/mL); +++++: very strong activity (28–33 mm); ++++: strong activity (22–27 mm); +++: medium activity (16–21 mm); ++: low activity (10–15 mm); +: very low activity (7–9 mm); −: no activity.

## Data Availability

Data are contained within the article.

## Supplementary Materials

The following supporting information can be downloaded at: https://www.mdpi.com/article/10.3390/pharmaceutics16010052/s1, Figure S1: HPLC Chromatogram of the used standard phenolic compounds; Figure S2: HPLC Chromatogram of the SP2N extract.
