# Supplementary material for: Comparative Antioxidant and Antimicrobial Activities of Several Conifer Needles and Bark Extracts
Source: Pharmaceutics. 2023 Dec 28;16(1):52. doi: 10.3390/pharmaceutics16010052 (PMC10821083; doi:10.3390/pharmaceutics16010052)
Supplement: Supplementary file 1 [file pharmaceutics-16-00052-s001.zip › pharmaceutics-2746278-supplementary.pdf]

SUPPLEMENTARY MATERIALS

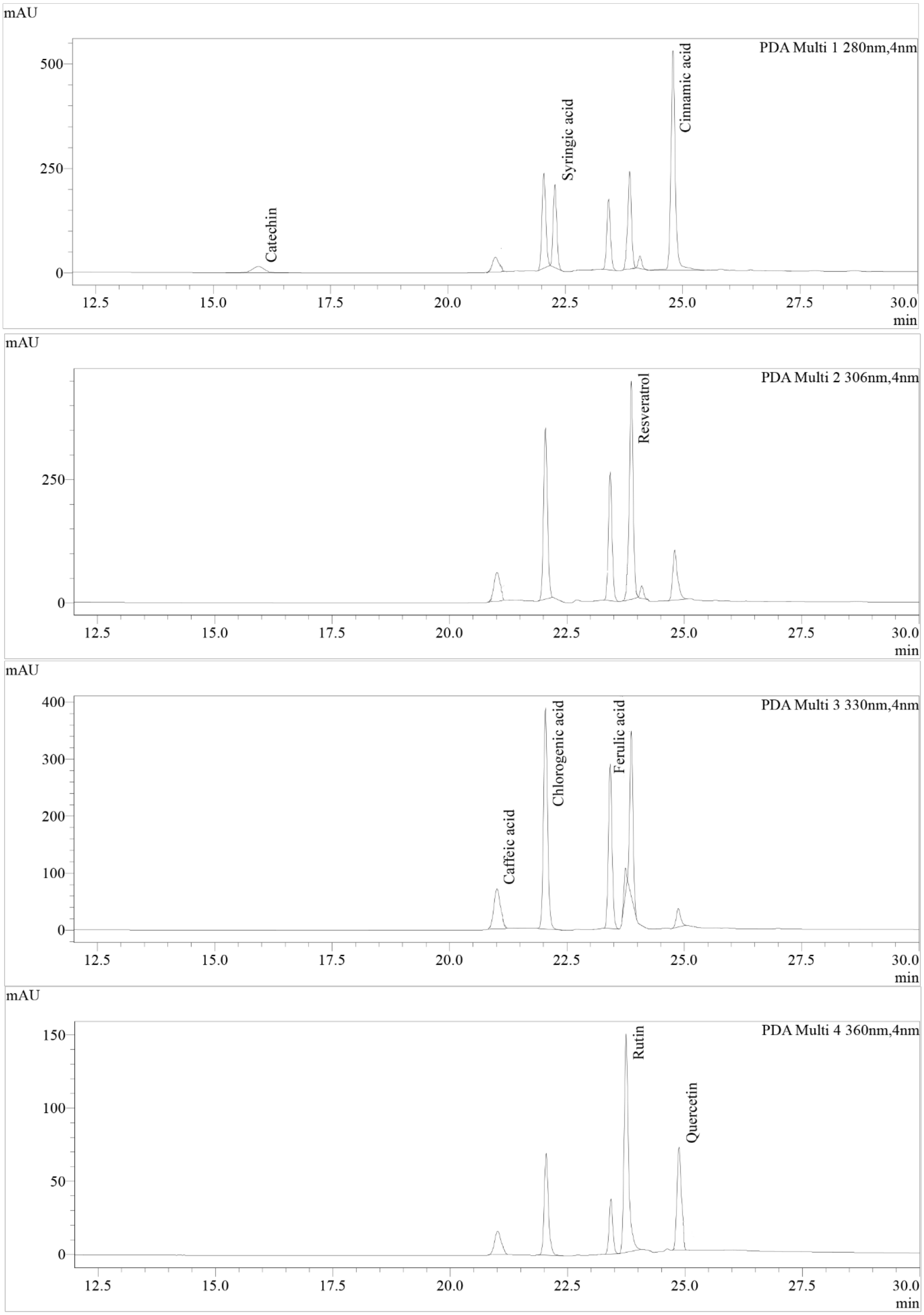

Figure S1. HPLC Chromatogram of the used standard phenolic compounds.

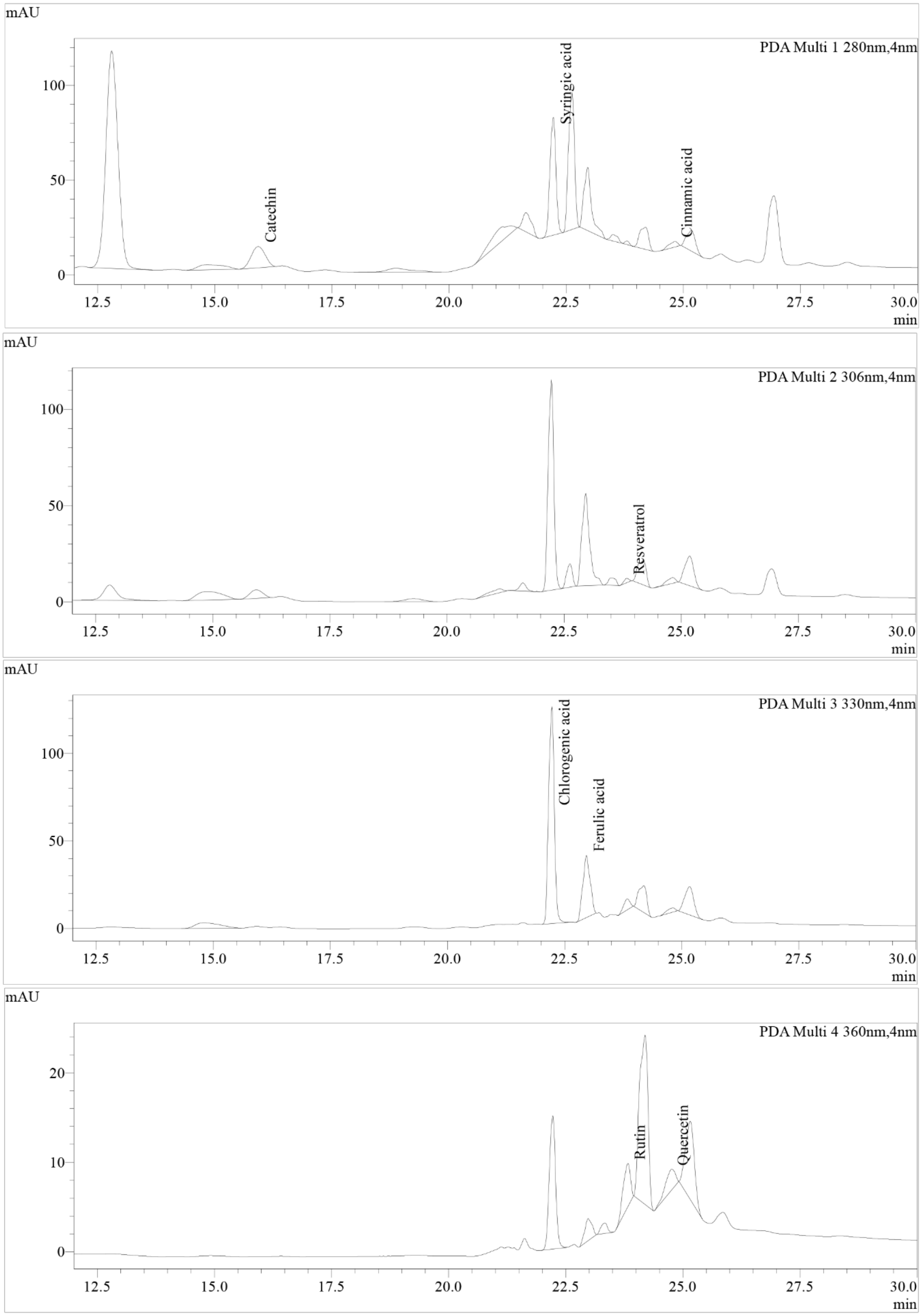

Figure S2. HPLC Chromatogram of the SP2N extract.
